# Chronic immune activation and inflammation as the cause of malignancy

**DOI:** 10.1054/bjoc.2001.1943

**Published:** 2001-08

**Authors:** K J O'Byrne, A G Dalgleish

**Affiliations:** Department of Oncology, University of Leicester Leicester; Division of Oncology, St George's Hospital Medical School, London, SW17 0RR, UK

**Keywords:** cell mediated immunity, humoral immunity, angiogenesis, cancer

## Abstract

Several chronic infections known to be associated with malignancy have established oncogenic properties. However the existence of chronic inflammatory conditions that do not have an established infective cause and are associated with the development of tumours strongly suggests that the inflammatory process itself provides the prerequisite environment for the development of malignancy. This environment includes upregulation of mediators of the inflammatory response such as cyclo-oxygenase (COX)-2 leading to the production of inflammatory cytokines and prostaglandins which themselves may suppress cell mediated immune responses and promote angiogenesis. These factors may also impact on cell growth and survival signalling pathways resulting in induction of cell proliferation and inhibition of apoptosis. Furthermore, chronic inflammation may lead to the production of reactive oxygen species and metabolites such as malondialdehyde within the affected cells that may in turn induce DNA damage and mutations and, as a result, be carcinogenic. Here it is proposed that the conditions provided by a chronic inflammatory environment are so essential for the progression of the neoplastic process that therapeutic intervention aimed at inhibiting inflammation, reducing angiogenesis and stimulating cell mediated immune responses may have a major role in reducing the incidence of common cancers. © 2001 Cancer Research Campaign http://www.bjcancer.com


*British Journal of Cancer* (2001) **85**(4), 473–483
© 2001 Cancer Research Campaign
doi: 10.1054/ bjoc.2001.1943, available online at http://www.idealibrary.com on IDEAL

http://www.bjcancer.com

Review

### Chronic immune activation and inflammation as the cause of malignancy

**KJ O'Byrne[1] and AG Dalgleish[2]**

[1]Department of Oncology, University of Leicester Leicester and [2]Division of Oncology, St George's Hospital Medical School, London SW17 0RR, UK

**Summary** Several chronic infections known to be associated with malignancy have established oncogenic properties. However the existence of chronic inflammatory conditions that do not have an established infective cause and are associated with the development of tumours strongly suggests that the inflammatory process itself provides the prerequisite environment for the development of malignancy. This environment includes upregulation of mediators of the inflammatory response such as cyclo-oxygenase (COX)-2 leading to the production of inflammatory cytokines and prostaglandins which themselves may suppress cell mediated immune responses and promote angiogenesis. These factors may also impact on cell growth and survival signalling pathways resulting in induction of cell proliferation and inhibition of apoptosis. Furthermore, chronic inflammation may lead to the production of reactive oxygen species and metabolites such as malondialdehyde within the affected cells that may in turn induce DNA damage and mutations and, as a result, be carcinogenic. Here it is proposed that the conditions provided by a chronic inflammatory environment are so essential for the progression of the neoplastic process that therapeutic intervention aimed at inhibiting inflammation, reducing angiogenesis and stimulating cell mediated immune responses may have a major role in reducing the incidence of common cancers. © 2001 Cancer Research Campaign http://www.bjcancer.com

**Keywords**: cell mediated immunity; humoral immunity; angiogenesis; cancer

Cancer arises as a result of a multi-step process leading from the initial benign transformation of cells through to overt invasive, metastatic disease (Lengauer et al, 1998; Raza, 2000; Vogelstein et al, 1988). This process takes many years to unfold and the length of time required strongly suggests that it does so against a background of rigorous controls aimed at preventing anarchic cell behaviour which would threaten the life of the individual, human or animal. It is likely that certain environments, coupled with a genetic predisposition, alter host cell susceptibility to carcinogenic insults. The degree of these effects may have a critical bearing on whether or not an individual exposed to a particular carcinogen develops malignant disease and, if so, the duration of exposure required for the tumour to occur. For example the majority of cigarette smokers never develop lung cancer. In contrast, many individuals do so from passive smoking only. Cigarette smoking results in chronic airway inflammation. However, the nature of the local inflammatory environment resulting from cigarette smoking is highly variable depending on polymorphic immune response genes in addition to a variety of anti-oxidant and DNA repair associated genes (Spitz et al, 1999). Here it is argued that chronic inflammation, resulting from infective and/or non-infective agents, may provide the ideal environment for the development of the cell changes that lead to cancer.

*Received 25 September 2000*
*Revised 30 April 2001*
*Accepted 30 May 2001*

*Correspondence to:* AG Dalgleish

### THE RELATIONSHIP BETWEEN CHRONIC IMMUNE ACTIVATION AND MALIGNANCY

Chronic activation of the immune system is, in itself, associated with the development of tumours such as lymphomas seen in HIV induced AIDS or chronic graft versus host disease (GVHD) (Dalgleish, 1992; Habeshaw et al, 1992). AIDS is also associated with the development of Kaposi's sarcoma (KS) a tumour caused, at least in part, by the herpes virus HHV-8 which in normal individuals rarely causes aggressive disease (Whitby and Boshoff, 1998). Other long standing infections associated with cancer include schistosomiasis and bladder cancer (Badawi et al, 1995), hepatitis B and C virus (HBV and HCV) and liver cancer (Imperial, 1999), Epstein-Barr virus and a range of lymphoproliferative and solid tumours including Burkitt's lymphoma (Kitagawa et al, 2000) and naso-pharyngeal cancer (Liu et al, 2000) and, more recently, *Helicobacter pylori* and stomach cancer (Williams and Pounder, 1999).

However, chronic inflammation, arising as a result of chronic exposure to a non-infective irritant, may also be associated with the development of malignant disease (Table 1). Chronic bronchitis and emphysema due to cigarette smoking are recognized risk factors for the development of lung cancer (Mayne et al, 1999). Carcinoma of the oesophago-gastric junction is one of the fastest rising cancers in the western world and is associated with chronic oesophagitis, including Barrett's oesophagus (Jankowski et al, 1999; McCann, 1999). Mesothelioma arises as a result of chronic exposure to asbestos fibres. The incidence in exposed individuals is increasing to such an extent that it is expected that 1 in 100 men in the UK born in the 1940s will die from the disease (Edwards et al, 2000). The association between chronic inflammatory bowel disease and cancer of the bowel is well established

(Kirk and Clements, 1999; Lewis et al, 1999). Furthermore, in the majority of colorectal tumours not associated with inflammatory bowel disease, histology shows that the precursor lesions, whether adenomas or polyps, are often inflammatory in nature (Higaki et al, 1999).

### THE INFLAMMATORY ENVIRONMENT ASSOCIATED WITH THE SUBSEQUENT DEVELOPMENT OF CANCER

Whereas the association between chronic immune activation and the development of cancer has been recognized for some years, only recently have we begun to understand the mechanisms underlying this phenomenon. The first concerns the nature of the local and systemic immune response seen in patients with chronic inflammatory conditions known to be associated with the development of malignant disease. Immune responses may be broadly divided into two categories – cell mediated immunity and humoral immunity. Cell mediated immunity (CMI) is associated with CD4 + T-lymphocytes which characteristically produce the cytokines interleukin(IL)-2, interferon-γ and tumour necrosis factor(TNF)-α (Thl-lymphocytes). Humoral immunity (HI) is associated with CD4+ T-lymphocytes which characteristically produce IL-4, IL-6 and IL-10 (Th2) (Mosmann and Coffman, 1989).

Recent experimental evidence suggests that exposure to a foreign antigen results in upregulation of the non-specific pro-inflammatory cytokines IL-1α and -β and the Th1 cytokines in inflammatory cells. Cyclooxygenase (COX)-1 and COX-2 are among the most important enzymes involved in the regulation of the immune response and play a key role in angiogenesis, the inhibition of apoptosis, and cell proliferation and motility. COX-1 is constitutively expressed by many cells. In contrast COX-2 is produced in response to exposure of epithelial, mesenchymal and inflammatory cells to inflammatory cytokines, (Taketo, 1998; Uotila, 1996; Vane et al, 1998), and infective and environmental agents known to be associated with the development of malignant disease such as *helicobacter pylori* infection (Sawaoka et al, 1998a), nicotine (Schror et al, 1998) and tobacco-specific nitrosamine 4-(methylnitrosamino)-4-(3-pyridyl)-1-butanone (NNK) (El-Bayoumy et al, 1999). Th2 cytokines such as IL-4 and IL-10, which inhibit the synthesis of Th1 cytokines by CD4+ T-helper lymphocytes, are produced in COX-2 expressing environments. These Th2 cytokines not only downregulate both proinflammatory/Th1 cytokines but also COX-2 expression itself (Della Bella et al, 1997; Subbaramaiah et al, 1997; Uotila, 1996; Vane et al, 1998). Chronic antigen exposure may drive a continuous cycle in which induced pro-inflammatory and Th1 cytokines upregulate COX-2 leading to chronic Th2 cytokine production and downregulation of the pro-inflammatory CMI response. In predisposed individuals such a cycle may eventually lead to the development of a predominant HI response environment (Figure 1).

### DOES SUCH AN ENVIRONMENT EXIST IN CANCER ASSOCIATED CHRONIC INFLAMMATORY CONDITIONS?

HIV induced AIDS, associated with the development of lymphoma and KS (Weiss and Loveday, 1999), and HIV infection are associated with a reduction in CMI (Westby et al, 1998). The enhanced immune activation seen in HIV disease is due to upregulation of HI responses, which may be important in

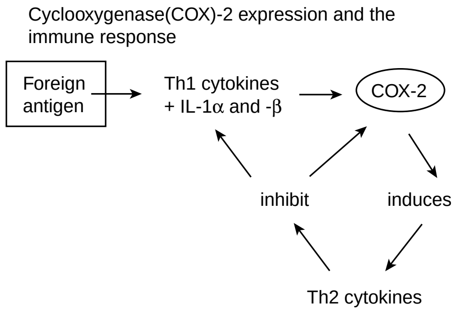

**Figure 1** Regulation of cyclo-oxygenase expression in the immune response. IL-1α = interleukin-1α; IL-1β = interleukin-1β; COX-2 = cyclooxygenase-2; Th1 = T-helper cell lymphocyte cytokine pattern 1 associated with cell mediated immune (CMI) responses; Th2 = T-helper cell lymphocyte cytokine pattern 2 associated with humoral immune (HI) responses. Chronic antigen exposure may drive a continuous cycle in which induced pro-inflammatory and Th1 cytokines upregulate COX-2 leading to chronic Th2 cytokine production and downregulation of the pro-inflammatory CMI response. In predisposed individuals such a cycle may eventually lead to the development of a predominant HI response environment

**Table 1** Factors predisposing to malignancy

| | Angiogenesis | ↓ CMI | ↑ HI |
|---|---|---|---|
| Carcinogens | | | |
| – Sunlight | + | + | + |
| – Nicotine | + | + | + |
| – Asbestos | + | + | + |
| Infections | | | |
| – HepB & C | + | + | + |
| – HPV | + | + | + |
| – HIV | + | + | + |
| – EBV | + | + | + |
| – SV-40 | + | + | + |
| Helicobacter pylori | + | + | + |
| Schistosomiasis | | | |
| Chronic inflammatory diseases | + | + | + |
| – IBD | | | |
| Growth facors / Immunosuppressive agents | | | |
| – IGF-1 | + | + | + |
| – TGF-b1 | + | + | + |
| (Cyclosporin A) | | | |
| Genetic mutations | | | |
| – p53 | + | + | ? |
| – Von Hippel Landau (VEGF) | + | + | ? |

 *© 2001 Cancer Research Campaign*

providing the necessary environment for EBV and HHV-8 to induce lymphoma and KS development respectively (Clerici and Shearer, 1994; Westby et al, 1998). Hence chronic immune activation associated with a predominant HI response may be a key factor contributing to the ideal environment necessary for viral driven tumours to occur. Where evaluated all chronic inflammatory infectious and non-infectious conditions associated with the development of cancer, including HBV and HCV associated chronic hepatitis and cirrhosis (Imperial, 1999), HPV associated cervicitis (al-Saleh et al, 1998; Le Buanec et al, 1999), schistosomiasis cystitis (Raziuddin et al, 1991), *helicobacter pylori* gastritis (Williams and Pounder, 1999) and asbestos exposure (Bielefeldt-Ohmann et al, 1996) are associated with similar immune changes (Table 1). The importance of the antigen induced pro-inflammatory and Th1 cytokine drive in the development of a Th2 predominant immune environment and the subsequent development of malignant disease is underlined by the observation that TNF deficient mice are resistant to skin carcinogenesis (Moore et al, 1999).

### RELATIONSHIP BETWEEN ANGIOGENESIS AND THE IMMUNE RESPONSE

The second aspect of chronic immune activation which may predispose to cancer concerns the relationship between the immune response and angiogenesis. Recent research has indicated that angiogenesis may play a key role in the development of early neoplastic lesions and subsequent malignancy (Bergers et al, 1999). There is considerable evidence that the angiogenesis associated with normal physiological processes such as wound healing occurs in the setting of a HI predominant environment (Folkman, 1995; Kodelja et al, 1997; Schaffer and Barbul, 1998; Singer and Clark, 1999). Co-culture experiments have shown that endothelial cell proliferation induced by HI stimulated macrophages is 3–3.5 times higher than that induced by CMI stimulated macrophages (Kodelja et al, 1997). The importance of Th2 cytokines in the angiogenic process is underlined by the observation that IL-6 knockout mice have an impaired capacity to regenerate normal hepatic tissue and to heal wounds (Gallucci et al, 2000; Wallenius et al, 2000). In parallel there is suppression of CMI, the latter presumably occurring so that damaged tissues such as skin and muscle do not become presented to the immune system as non-self and induce an autoimmune response to healing or healed tissues (Schaffer and Barbul, 1998; Singer and Clark, 1999). In contrast to HI immune response induced angiogenesis, CMI immune responses tend to inhibit angiogenesis (Watanabe et al, 1997). The inverse relationship between CMI suppression and enhanced angiogenesis is seen not only in wound healing but also in other normal physiological conditions such as ovulation and pregnancy (Bergers et al, 1999; Folkman, 1995; Piccinni et al, 1998; Richards et al, 1995).

Unlike normal physiological processes, the factors that suppress CM1 and switch on angiogenesis persist in many established chronic infection/inflammatory states, particularly those conditions discussed earlier that are associated with the development of malignant disease (Table 1). If this state occurs for several years then random mutations in the cells of the affected tissues, caused by carcinogens or unregulated proliferation, would occur in an immunologically tolerant environment. Phenotypic changes, e.g. proteins resulting from mutations in the *ras* oncogene, which would normally be detected by cytotoxic lymphocytes, may escape immune surveillance thus allowing another step in the stochastic progression towards malignancy to occur (Gjertsen et al, 1997). Indeed, it is so important for this environment to be maintained that developing neoplastic cell clones evolve to mimic this state in order to progress and metastasize.

Again induction of COX-2 may be central to the development of an angiogenic environment in many of the conditions leading to the subsequent development of malignancy. COX-2 expressing tumour cells are associated with the production of a number of angiogenic growth factors and the synthesis and activation of matrix metalloproteinases favouring tumour invasion and angiogenesis (Tsujii et al, 1997; Tsujii et al, 1998; Takahashi et al, 1999).

### THE IMMUNE RESPONSE AND ANGIOGENESIS IN MALIGNANT DISEASE

Where studied, all malignancies are associated with suppression of CMI (Lee et al, 1997; Maraveyas et al, 1999; Pettit et al, 2000). Indeed, even early stage Dukes A colorectal patients have a suppressed CMI response which reverts to normal upon surgical removal of the tumour (Heriot et al, 2000). The dramatic nature of this response suggests that the HI predominant, pro-angiogenic environment is an absolute requirement for further disease progression. If a tumour requires shielding from immune surveillance to grow, then it will employ such tactics in order to metastasize and seed into other tissue sites. The number of documented strategies employed by cancers to evade the immune response continues to expand. These include the downregulation of HLA and costimulatory molecules, production of immunosuppressive factors and upregulation of immune cell apoptosis inducing molecules such as Fas L (Doherty et al, 1994; Ganss and Hanahan, 1998; Garrido et al, 1993; Gorter and Meri, 1999; Melief and Kast, 1991; Strand and Galle, 1998; Pettit et al, 2000). In keeping with the avoidance of immune surveillance, COX-2 is constitutively upregulated in a range of premalignant lesions and tumours including those arising from the colon and rectum, stomach, lung, pancreas, head and neck and breast (Murata et al, 1999; Koshiba et al, 1999; Mestre et al, 1999; Molina et al, 1999; Wolff et al, 1998; Huang et al, 1998; Taketo, 1998; Tsujii et al, 1997; Uotila, 1996; Vainio and Morgan, 1998; Vane et al, 1998).

Angiogenesis, the formation of new blood vessels from an existing vasculature, is essential for tumour growth beyond 1–2 mm in diameter. This process occurs in all tumours and is under the regulation of pro-angiogenic factors including Th2 cytokines such as IL-6 and vascular endothelial growth factor (VEGF). The intensity of the angiogenic process, as assessed by microvessel counting methods, correlates with primary tumour growth, invasiveness, and metastatic spread of disease (Folkman, 1995; O'Byrne et al, 2000)

If the immune surveillance evading mechanisms and the angiogenesis observed in malignant disease processes are indeed central to tumour growth and metastasis it would appear logical to suppose that the environment within which malignant transformation occurs might have similar characteristics. This contention is supported by the observation that all of the chronic immune activated disease processes studied to date which predispose to the development of malignant disease are associated with a suppressed CMI and an upregulated HI, proangiogenic environment as discussed (Table 1).

### CHRONIC INFLAMMATION AND INHIBITION OF APOPTOSIS

In both non-neoplastic and neoplastic cells COX-2 is associated with cell proliferation (Tsuji et al, 1996) (McGinty et al, 2000) and inhibition of apoptosis at least in part through the induction of

© 2001 Cancer Research Campaign

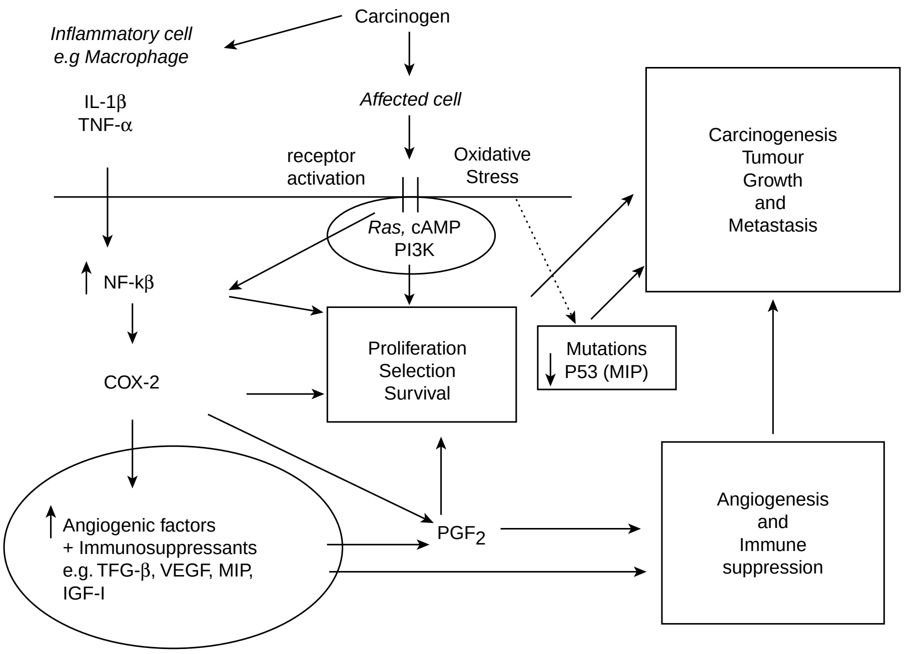

**Figure 2** Sequence of events which occurs in those chronic inflammatory conditions known to predispose to the development of cancer. cAMP = cyclic Adenosine monophosphate; PI3K = phosphatidylinositol-3-kinase; IL-1β = interleukin-1β; TNF-α = tumour necrosis factor-α; NF-kβ = nuclear factor-kβ; COX-2 = cyclo-oxygenase-2; TGF-β = transforming growth factor-β; VEGF = vascular endothelial growth factor; IGF-I = insulin-like growth factor-I; MIP = macrophage inhibitory factor; $PGE_2$ = prostaglandin-$E_2$. Exposure to a carcinogen known to induce chronic inflammation/immune activation may lead to changes both in the microenvironment and within the cells of the affected tissue pre-disposing them to the subsequent development of malignancy bcl-2 (Tsujii and DuBois, 1995). There is increasing evidence that exposure to carcinogens such as ultraviolet B light (Athar et al, 2001), the tobacco specific carcinogen 4-(methylnitrosamino)-1-(3-pyridyl)-1-butanone (NNK) (El-Bayoumy et al, 1999) and nicotine (Saareks et al, 1998), and helicobacter pylori (Konturek et al, 2000; Sawaoka et al, 1998a) leads to the upregulation of COX-2 in the affected tissue

Carcinogens act, at least in part, through the induction of growth factors and activation of growth factor receptors. An example of a growth factor known to be induced by carcinogenic insults is the epidermal growth factor receptor (EGFR) which is upregulated in many malignant and pre-malignant conditions including those of the lung (Cox et al, 2000). In the keratinocyte ultraviolet (UV)-B irradiation have been demonstrated to induce EGFR phosphorylation and activation of the extracellular-regulated kinase 1 and 2 (ERK1/2) pathways. This occurs secondary to oxidative stress, itself caused by UV-B generated $H_2O_2$. Pretreatment of the cells with the specific EGFR inhibitor PD153035 followed by UVB induced $H_2O_2$ production reduces the clonogenic potential of keratinocytes. This reduced proliferation is associated with increased apoptosis and cell death (Peus et al, 2000). Likewise, asbestos fibres result in upregulation and phosphorylation of EGFR in mesothelial cells and, after 48 h, selection of cells which survive the initial apoptotic effects of the fibres and proliferate (Faux et al, 2000). These results indicate that EGFR phosphorylation induces downstream signaling pathways which play a fundamental role in regulating cell survival mechanisms following oxidative stress (Peus et al, 2000). Given these results it is therefore not surprising that recent data indicate that EGFR activation results in COX-2 expression (Mestre et al, 1999).

The pathways involved in EGFR upregulation of COX-2 and cell survival remain to be fully elucidated but current evidence suggests an important role for nuclear factor (NF)-kβ, a transcription factor important in the regulation of a number of genes intrinsic to inflammation and cell proliferation (Thanos and Maniatis, 1995). EGFR phosphorylation activates phosphatidylinositol 3-kinase (PI3K) (Hu et al, 1992). Activation of PI3K generates phosphotidylinositol-3, 4-P2 which in turn recruits and activates the downstream serine/threonine kinase, Akt. Activated Akt phosphorylates specific targets such as Bad (del Peso et al, 1997) and procaspase-9 (Cardone et al, 1998) with the result of promoting cell survival. PI3K is involved in the activation of the transcription factor NF-kβ (Beraud et al, 1999). Activation of NF-kβ *via* the Akt signalling pathway is involved in cell survival and resistance to apoptosis induced by TNF-a (Zhou et al, 2000). Carcinogenic asbestos fibres induce NF-kβ activation in Simian Virus (SV)-40 transformed mesothelial (MET 5A) cells and this is linked to cell proliferation (Faux and Howden, 1997; Janssen et al, 1997; Faux et al, 2000). Using gel mobility shift assays pretreatment with the selective EGFR tyrosine kinase inhibitor, PKI166 (Novartis Pharmaceuticals), has been demonstrated to inhibit the DNA binding of NF-kβ. Both PKI166 and NF-kβ decoy proteins reduce cell viability demonstrating the importance of this pathway in cell proliferation and survival (Faux et al, 2001). The NF-kβ binding motif is found in the promoter region of the COX-2 gene (Du Bois et al, 1998). In keeping with the important role of pro-inflammatory cytokines and angiogenic growth factors in the carcinogenic process IL-1β and bFGF combined with EGF have been shown to enhance the induction of COX-2 (Majima et al, 1997; Yucel-Lindberg et al, 1999).

Macrophage inhibitory factor (MIF) is released during inflammatory states and has recently been shown to repress the transcription activity of p53 and its downstream targets of p21 and bax, thereby having a marked anti-apoptotic effect (Cordon-Cardo and Prives, 1999; Hudson et al, 1999). Hence, inflammation can actively contribute to the perturbation of probably the single most

 *© 2001 Cancer Research Campaign*

important cell regulatory pathway in cancer control, thus further preventing the body's own cellular defences from reacting to a mutagenic or stochastic event. P53 also plays a central role in the regulation of angiogenesis, in part through induction of the anti-angiogenic factor thrombospondin (Dameron, 1994), and the mediation of Th1 cytokine induced cytotoxicity (Kano et al, 1997; Yeung and Lau, 1998). As such, loss of p53 would also facilitate angiogenesis and be associated with an impaired CMI response. A number of other cytokines, including TNF and GM-CSF, and growth factors such as insulin-like growth factor (IGF)-I have also been implicated directly in cell cycle and angiogenic control, which, under the appropriate circumstances, could lead to enhanced carcinogenesis (O'Byrne et al, 2000).

Therefore, as well as inducing angiogenesis and a HI predominant immune response, chronic inflammation may also lead to inhibition of apoptosis in the affected cells. The reactive oxygen species induced by carcinogens and the formation of carcinogenic metabolites produced by the inflammatory process, e.g. malondialdehyde resulting from the metabolism of arachidonic acid by COX-2 (Subbaramiah et al, 1997), may lead directly to DNA damage and subsequent mutations. Under these circumstances, and through microenvironmental selection pressures (Pettit et al, 2000), mutated cell populations may not only survive but thrive, transform and eventually take on a malignant phenotype (Figure 2).

### ONCOGENIC VIRUSES AND CANCER

Whilst it is clear that viral infections such as HBV and HCV do not cause cancer unless chronic inflammation occurs, and then only after many years have passed, the situation is often less clear-cut for other oncogenic viruses. For example human papilloma virus (HPV) is clearly linked to the development of cancer of the cervix. Elegant molecular mechanisms have demonstrated that the E6 and E7 human papilloma virus proteins bind to and inhibit the activity of the P53 and retinoblastoma (rb) tumour suppressor gene proteins (Dalgleish, 1991). However, only in the past few years is evidence accumulating that persistent HPV infection is associated with a chronically immune activated state locally in the cervix and, perhaps also systemically. While over 25% of females are infected at ages 19–25, less than 5% remain infected over 35 years of age. It is possible that these observations may be explained by differences in the methodology for detecting the virus and by sampling error. Nonetheless, recent studies indicate that failure to clear the viral infection results in persistent inflammation with chronic cervicitis and an increased risk of developing cancer of the cervix (Cerqueira et al, 1998; White et al, 1992; Hsieh et al, 1999). This may be contributed to by co-infection with chlamydia. HPV and chlamydial infection have been shown to be associated with increased proliferation of the ectocervical epithelium. This is associated with reduced apoptosis (Vaganova, 2000). HPV is associated with increased circulating levels of IL-2 soluble receptor, a non-specific marker of inflammation, in a proportion of otherwise normal infected individuals. The number of infected individuals with elevated levels rises significantly with the development of CIN and subsequently invasive cervical cancer (Hildesheim et al, 1997; Ung et al, 1999). A recent longitudinal study of HPV infected patients, in which the virus was detected using the hybrid capture II assay, demonstrated that a proportion of patients found to have persistent infection after 2–3 assessments developed cervical intraepithelial neoplasia (CIN). In contrast those individuals found to have cleared the infection did not develop any CIN lesions (Clavel et al, 2000). Several studies have indicated that clearance of the virus is associated with the development of a CMI response including IL-2 Th1 responses to the c-terminal domain of the HPV-16 E2 protein (Bonktes et al, 1999), upregulation of IFN-$\gamma$ in exfoliated cervical cells (Scott et al, 1999) and an IgA antibody response (Bonktes et al, 1999). Indeed, the presence of a hypersensitivity reaction, an indicator of a CMI response, to the HPV-16 oncoprotein E7 is associated with the subsequent regression of CIN lesions (Hopfl et al, 2000). In contrast in patients with active CIN, Th2 immune responses predominate with an increased IL-10/IL-12 ratio in whole blood supernatants (Jacobs et al, 1998). CD3+ DR+ antigen peripheral blood T-lymphocytes, and the level of CD4+ T-cells have been shown to decrease while the level of the CD8+ cells increase in women with CIN as it progresses to frank carcinoma-in-situ. In contrast the number of B cells remains unchanged (Spivak et al, 1999).

Epstein–Barr virus (EBV) is associated with Burkitt's Lymphoma (BL) in Africa where it arises in children whose immune system is chronically activated by malaria (de The G,1993). Likewise, EBV associated nasopharyngeal cancer (NPC) occurs in Asia (Liu et al, 2000) where it is prevalent only amongst people who are exposed to fish treated by a smoke curing process which may also cause local inflammation (Zheng et al, 1999). Although the majority of the western population is infected with EBV only a minority develop associated malignancies, in particular, lymphomas (Yamamoto et al, 1999; Mauray et al, 2000). Other malignancies associated with EBV virus infection include squamous oesophageal (Wang et al, 1999) and gastric cancer (Takada, 2000). However, there is increasing evidence that even under these circumstances, in which immune activation may not be readily apparent, EBV gene products may contribute to the inhibition of apoptosis, increased angiogenesis, suppression of CMI responses and a HI predominant environment. EBV transforming gene product BARF1 (zur Hausen et al, 2000) and EBV BHRF1, a homologue of the anti-apoptotic factor bcl-2, have been detected in EBV associated tumours (Liu et al, 2000). BL and natural killer/T-cell lymphomas growth is supported by EBV-encoded poly(A)(–) RNA through induction of IL-10 (Kitagawa et al, 2000). In nasal type extranodal cutaneous natural killer or T(NK/T)-cell lymphoma recent evidence indicates that expression of the latency associated EBV genes BHRF1, encoding the bcl-2 homologue, and BCRF1, encoding viral IL-10 favour tumour growth (Xu et al, 2001). EBV-encoded latent membrane protein 1 (LMP1) activation of the p38 mitogen-activated protein kinase pathway has been demonstrated to co-regulate IL-6 and IL-8 (a pro-angiogenic, HI associated chemokine) production (Eliopoulos et al, 1999, Mauray et al, 2000). Furthermore a strong association has been found between LMP1 expression and MMP-9, and metastasis in NPC (Horikawa et al, 2000). In keeping with the crucial role of pro-inflammatory cytokines in the pathogenesis of malignant disease increased IL-1$\alpha$ and IL-1$\beta$ expression has been observed in primary NPC and metastases compared to control tissues and this observation was found to correlate with EBV-encoded viral IL-10 transcript (Huang et al, 1999).

Simian virus-40 (SV-40) is a virus implicated in the pathogenesis of a number of malignancies including mesothelioma, bone tumours, sarcomas, ependymomas and choroid plexus tumours, SV-40 virus oncoprotein, SV-40 large T antigen, binds p53 protein and each of the retinoblastoma family proteins, pRb, p107, and pRb2/p130 (Carbone et al, 1999; De Luca et al, 1997). Through

*© 2001 Cancer Research Campaign*

these effects the oncoprotein would facilitate angiogenesis (Dameron, 1994), reduce Th1 cytokine mediated cytotoxicity (Kano et al, 1997; Yeung and Lau, 1998) and inhibit apoptosis (Hudson et al, 1999) thereby predisposing the infected individual to the development of malignant tumours in infected tissues.

These data suggest that exposure to human oncogenic viruses rarely causes cancer unless chronic immune activation or inflammation is also present. Even in those quiescent infectious states where chronic inflammation may not be readily apparent the local changes induced by viral oncogenes can result in a HI predominant, proangiogenic, anti-apoptotic environment conducive to the development of malignant disease. The same may also be true for cancers associated with non-infectious carcinogenic insults, including cigarette smoking and asbestos exposure, which themselves cause inflammation.

### CANCER CONTROL AND CHAOS

Cancer and its environment, including the immune response and cellular control pathways, represent a complex system of interacting factors. Based on the chaos theory, the factors involved in the development of cancer represent non-linear or chaotic processes (Coffey, 1998). Chaos is associated with unpredictability, because too many acting forces are present in the system, and order, in that such complex processes occur against the background of major attractors. In the case of the immune system the simplified concept of CMI and HI immune responses represent two attractors which in addition to being self-regulatory with bilateral feedback pathways are affected by certain outside forces such as chronic infections or chemical and/or physical irritants (Dalgleish, 1999).

The major regulatory pathway factors including p53, p21 and bcl-2, themselves major attractors (or cellular policemen) in their own right, are also affected. Once significantly perturbated further stochastic oncogenic effects can progress. The relevance of this concept is that treatments that return the attractors to normal may be able to have significant indirect anti-cancer activity if applied before the cancer has progressed too far. With regards to the immune response, modulation of Th1/Th2 ratios towards a CMI predominant phenotype using relevant vaccine/cytokine protocols, COX-2 inhibitors and other anti-angiogenic agents may be able to shift the balance away from tumour growth and progression towards inhibition of tumour cell proliferation and, indeed, tumour regression. This may be what is occurring in those patients with solid tumours such as melanoma or renal cell cancer whose tumours regress following non-specific vaccination with BCG or similar agents and/or IL-2 therapy (Browning, 1996; Dalgleish, 1999; Vile and Dalgleish, 1996).

### RELEVANCE TO CONTROL AND PREVENTION

Recent research has clearly demonstrated that non-steroidal anti-inflammatory drugs (NSAIDs) and specific COX-2 inhibitors can inhibit solid tumour cell proliferation in vitro and in vivo. These agents may also prevent haematogenous spread of malignancy provided the disease over-expresses COX-2, and suppress angiogenesis and tumour growth in xenografts (Hida et al, 1998; Molina et al, 1999; Sawaoka et al, 1998b; Sawaoka et al, 1998c; Sawaoka

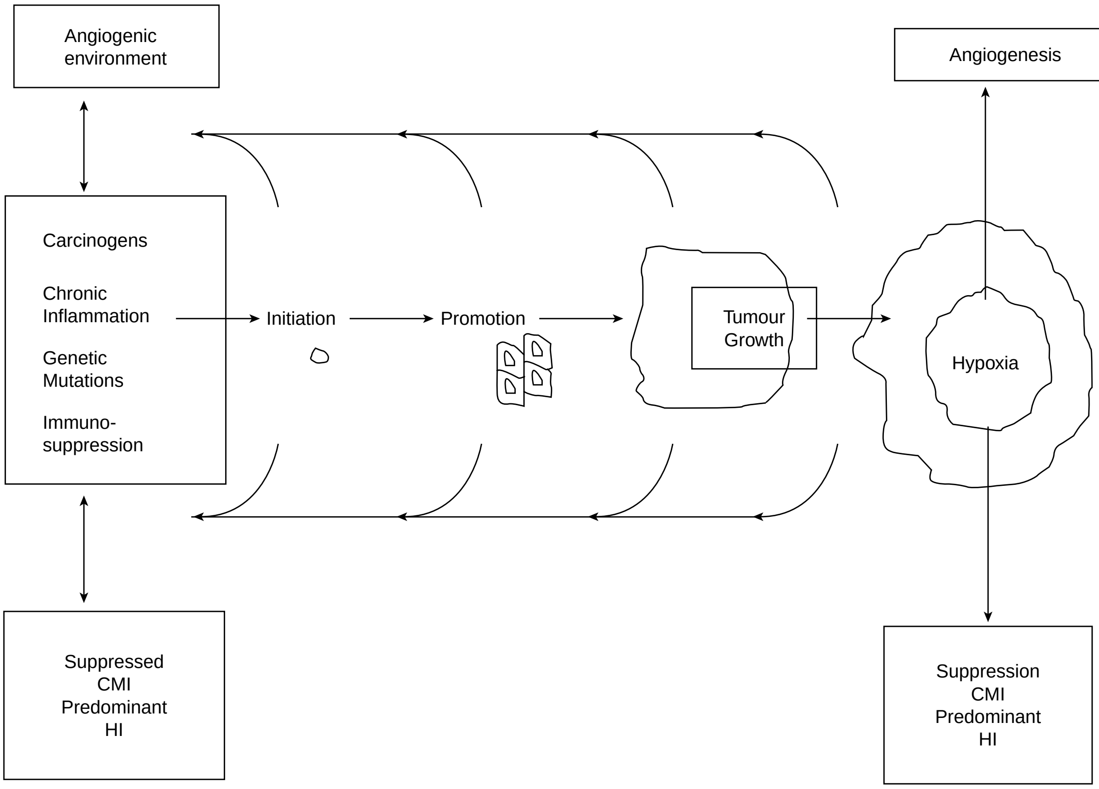

**Figure 3** Chronic immune activation or similar environments may support the development and selection of cells capable of transforming and eventually assuming a malignant phenotype. With progressive stages the tumour itself becomes the predominant factor downregulating cell mediated immune (CMI) and upregulating humoral immune (HI) responses and inducing angiogenesis. The situation is further exacerbated by tumour induced hypoxia which likewise inhibits CMI and induced both a predominant HI immune environment and microvessel formation (O'Byrne et al, 2000)

 *© 2001 Cancer Research Campaign*

et al, 1999; Tomozawa et al, 1999; Tsuji et al, 1996; Gately, 2000). Of greater relevance to the contention that chronic immune activation plays a central role in carcinogenesis is the finding that non-specific and specific COX-2 inhibitors may inhibit malignant transformation in a variety of experimental in vivo models including those for breast and lung cancer (Duperron and Castonguay, 1997; Lala et al, 1997; Yao et al, 2000).

There is already considerable evidence in the literature that long-term exposure to aspirin and other NSAIDs reduces the incidence of oesophageal, gastric, colorectal, bladder and lung cancer (Castelao et al, 2000; Funkhouser and Sharp, 1995; Giovannucci et al, 1995; Giovannucci et al, 1994; Langman et al, 2000; Paganini-Hill, 1994; Schreinemachers and Everson, 1994; Study, 1992; Thun et al, 1991; Akre et al, 2001).

Aspirin has a number of effects on cancer cells in vitro such as enhancing apoptosis. However, it is its effect on the inhibition of the cyclo oxygenases and, as a result, prostaglandin synthesis and its subsequent effect on the immune response that is of most interest because NSAIDs have been shown to prevent tumour mediated immunosuppression and angiogenesis (Grinwich and Plescia, 1977; Subbaramaiah et al, 1997; Taketo, 1998; Tsujii et al, 1998; Vane et al, 1998).

In order to prove that NSAIDs and selective COX-2 inhibitors have a role in chemoprevention one would want to conduct randomized studies in thousands of people for several years using an anti-inflammatory agent versus placebo.

### Not treating cancer one by one

There is a need to identify individuals at high risk of developing malignant disease and reduce the ‘promoter’ exposure. In the absence of obvious factors, such as cigarettes, the reduction of inflammation, inhibition of angiogenesis and restoration of CMI predominant immune response should be primary goals. Following the success of tamoxifen and raloxifene in reducing the incidence of breast cancer, particularly in at risk patients (reviewed in Savanthanan and O’Byrne, 2001) this approach should become a high priority in the chemoprevention of tumours at other sites. If successful chemopreventive strategies may result in a reduction of the number of patients requiring one to one treatment for established malignant disease at a later time. Trials could test the relevant contributions of anti-inflammatory, anti-angiogenic and CMI immune stimulatory agents through their use as single agents or in combination as chemopreventive and adjuvant therapies, and in the management of established inoperable/metastatic malignant disease.

### EXCEPTIONS THAT PROVE THE RULE?

Ever since the possible association between chronic infection and the development of cancer was first recognized, it was felt that this may be relevant to only a minority of cancers. However, as previously discussed, many tumours not associated with chronic infections such as lung, oesophageal and bowel cancers also fit this model. It therefore behoves us to ask the question ‘what tumours do not appear to be relevant to this model?’. Ironically the two immunologically associated tumours, melanoma and renal cell cancer as well as the endocrinologically sensitive tumours (breast and prostate) do not readily fit and neither do germ cell tumours and those which are clearly associated with inherited genetic defects.

However, in the case of melanoma, not only is there a genetic susceptibility (fair skinned, freckles, ginger hair, moles) but also a history of recurrent severe sunburn, a phenomenon associated with Th1 immune suppression and angiogenesis (Pamphilon et al, 1991). Renal cell cancer may represent a stochastic genetic evolution on the susceptible gene pool not requiring immune activation or systemic immunosuppression. However, the majority of renal cell carcinomas have associated mutations in the Von Hippel Landau tumour suppressor gene. This is associated with upregulation of the angiogenic growth factor VEGF (Fleming, 1999). VEGF has recently been recognized to suppress dendritic cell function, an important mediator of CMI (Gabrilovich et al, 1999). Interestingly, recent work has also demonstrated that insults to the kidney, such as dehydration, may be associated with an increase in cyclo-oxygenase-2 levels which could result in locally suppressed responses and upregulation of pro-angiogenic growth factors such as VEGF (Yang et al, 1999).

The endocrine sensitive tumours, such as breast and prostate cancer, have been shown to occur more frequently in the presence of chronic inflammatory histologies (mastitis and prostatitis respectively) (Monson et al, 1976; Nakata et al, 1993; Prince and Hildreth, 1986). In a recent study the EBV genome has been detected in the tumour cells of a significant proportion of patients with breast cancer but not in surrounding normal breast tissue. The detection of EBV has also been associated with metastatic spread of the disease to lymph nodes (Bonnet et al, 1999). As discussed earlier incorporation of EBV genes into the human genome may predispose affected tissue to the development of malignant disease due to inhibition of apoptosis, induction of cell transformation, enhanced tumour cell invasiveness and the induction of a Th2 predominant, pro-angiogenic environment through the release of IL-6, IL-8 and IL-10. In the case of prostatitis and prostate cancer diagnostic confusion is frequent (Jung et al, 1998). PCR detection of aseptic bacterial infection has been found in chronic prostatitis (Keay et al, 1999) and acute prostatitis can result in dissemination of prostate epithelial cells (Dumas et al, 1997). Furthermore, recent work has clearly demonstrated that the risk of biochemical relapse following radical prostatectomy is increased in patients with high grade inflammation surrounding malignant glands (Irani et al, 1999). It is thus surprising that a possible connection between prostatitis and prostate cancer has only rarely been commented on (De Marzo et al, 1999). The endocrine system also interacts directly with the immune response with Th1 and DHEA steroids counter-regulating Th2 and cortisol steroid pathways which may indicate that the inflammatory components may be more subtle than in non-endocrine tumours (Rook et al, 1994).

Recent observations indicate an important role for growth factors in the aetiology of breast and prostate cancer. Prospective clinical studies have demonstrated that high ‘normal’ IGF-1 levels are associated with the development of these tumours. IGF-I is an angiogenic growth factor which, in high levels, may suppress CMI responses (reviewed in O’Byrne et al, 2000). Recently, IGF-II acting through the IGF-I receptor has been shown to induce COX-2 and $PGE_2$ expression in colorectal cells indicating a possible mechanism by which IGF growth factors may play a key role in the initiation of malignant disease (Di Popolo et al, 2000).

Testicular cancer is increasing rapidly in incidence. The disease is very sensitive to treatment with cytotoxic chemotherapeutic agents and the majority of cases treated are cured. It is postulated that the chemosensitivity is due to the low incidence of p53

*© 2001 Cancer Research Campaign*

abnormalities detected in the disease. Given that it arises in an immunologically privileged site the comments regarding p53 and the interaction with the immune system previously mentioned are particularly compelling (Guillou et al, 1996). With over 300 different types of cancer there are bound to be alternative pathogenic pathways. However, if the chronic immune activation hypothesis applies then, at the very least, a significant proportion of all cancers may be amenable to modulation with combination therapies including anti-inflammatory, anti-angiogenic and CMI/Th1 enhancing agents. The question remains, is there any reason not to do these studies now?

### ACKNOWLEDGEMENTS

Dr Kenneth O'Byrne is supported by the Institute of Cancer Studies, Leicester and Prof. A G Dalgleish, by the Cancer Vaccine Campaign, UK.

### REFERENCES

Aker K, Ekstrom AM, Signorello LB, Hansson L-E and Nyren O (2001) Aspirin and risk of gastric cancer: a population-based case-control study in Sweden. *Br J Cancer* **84**: 965–968

al-Saleh W, Giannini SL, Jacobs N, Moutschen M, Doyen J, Boniver J and Delvenne P (1998) Correlation of T-helper secretory differentiation and types of antigen-presenting cells in squamous intraepithelial lesions of the uterine cervix. *J Pathol* **184**: 283–290

Athar M, An KP, Morel KD, Kim AL, Aszterbaum M, Longley J, Epstein EH Jr and Bickers DR (2001) Ultraviolet B(UVB)-induced cox-2 expression in murine skin: an immunohistochemical study. *Biochem Biophys Res Commun* **280**: 1042–1047

Badawi AF, Mostafa MH, Probert A and O'Connor PJ (1995) Role of schistosomiasis in human bladder cancer: evidence of association, aetiological factors, and basic mechanisms of carcinogenesis. *Eur J Cancer Prey* **4**: 45–59

Beraud C, Henzel WJ and Baeuerle PA (1999) Involvement of regulatory and catalytic subunits of phosphoinositide 3-kinase in NF-kB activation. *Proc Natl Acad Sci* **96**: 429–434

Bergers G, Javaherian K, Lo KM, Folkman J and Hanahan D (1999) Effects of angiogenesis inhibitors on multistage carcinogenesis in mice. *Science* **284**: 808–812

Bielefeldt-Ohmann H, Jarnicki AG and Fitzpatrick DR (1996) Molecular pathobiology and immunology of malignant mesothelioma. *J Pathol* **178**: 369–378

Bonnet M, Guinebretiere JM, Kremmer E, Grunewald V, Benhamou E, Contesso G and Joab I (1999) Detection of Epstein-Barr Virus in Invasive Breast Cancers. *J Natl Cancer Inst* **91**: 1376–1381

Bontkes HJ, de Gruijl TD, Bijl A, Verheijen RH, Meijer CJ, Scheper RJ, Stern PL, Burns JE, Maitland NJ and Walboomers JM (1999) Human papillomavirus type 16 E2-specific T-helper lymphocyte responses in patients with cervical intraepithelial neoplasia. *J Gen Virol* **80**: 2453–2459

Bontkes HJ, de Gruijl TD, Walboomers JM, Schiller JT, Dillner J, Helmerhorst TJ, Verheijen RH, Scheper RJ and Meijer CJ (1999) Immune responses against human papillomavirus (HPV) type 16 virus-like particles in a cohort study of women with cervical intraepithelial neoplasia. II. Systemic but not local IgA responses correlate with clearance of HPV-16. *J Gen Virol* **80**: 409–417

Browning MDA (1996) *Introduction and Historical perspective*. In: *Tumour Immunology*. Cambridge University Press: Cambridge

Carbone M, Rizzo P, Gromley PM et al (1997) Simian virus-40 large-T antigen binds p53 in human mesotheliomas. *Nature Med* **8**: 908–912

Cardone MH, Roy N, Stennicke HR, Salvesen GS, Franke TF, Stanbridge E, Frisch S and Reed JC (1998) Regulation of cell death protease caspase-9 by phosphorylation. *Science* **282**: 1318–1321

Castelao JE, Yuan JM, Gago-Dominguez M, Yu MC and Ross RK (2000) Non-steroidal anti-inflammatory drugs and bladder cancer prevention. *Br J Cancer* **82**: 1364–1369

Cerqueira EM, Santoro CL, Donozo NF, Freitas BA, Pereira CA, Bevilacqua RG and Machado-Santelli GM (1998) Genetic damage in exfoliated cells of the uterine cervix. Association and interaction between cigarette smoking and progression to malignant transformation? *Acta Cytol* **42**: 639–649

Clavel C, Masure M, Levert M, Putaud I, Mangeonjean C, Lorenzato M, Nazeyrollas P, Gabriel R, Quereux C and Birembaut P (2000) Human papillomavirus detection by the hybrid capture II assay: a reliable test to select women with normal cervical smears at risk for developing cervical lesions *Diagn Mol Pathol* **9**: 145–150

Clerici M and Shearer GM (1994) The Th1-Th2 hypothesis of HIV infection: new insights. *Immunol Today* **15**: 575–581

Coffey DS (1998) Self-organization, complexity and chaos: the new biology for medicine [see comments]. *Nat Med* **4**: 882–885

Cordon-Cardo C and Prives C (1999) At the crossroads of inflammation and tumorigenesis [comment]. *J Exp Med* **190**: 1367–1370

Cox G, Jones LJ and O'Byrne KJ (2000) MMP-9 and the epidermal growth factor signal pathway in operable non-small cell lung cancer. *Clin Cancer Res* **6**: 2349–2355

Dalgleish A (1999) The relevance of non-linear mathematics (chaos theory) to the treatment of cancer, the role of the immune response and the potential for vaccines. *Q J Med* **92**: 347–359

Dalgleish AG (1991) Viruses and cancer. *Br Mèd Bull* **47**: 21–46

Dalgleish AG (1992) The pathogenesis of AIDS: classical and alternative views. *J R Coll Physicians Lond* **26**: 152–158

Dameron KM, Volpert OV, Tainsky MA and Bouck N (1994) Control of angiogenesis in fibroblasts by p53 regulation of thrombospondin-1. *Science* **265**: 1582–1584

De Luca A, Baldi A, Esposito V, Howard CM, Bagella L, Rizzo P, Caputi M, Pass HI, Giordano GG, Baldi F, Carbone M and Giordano A (1997) The retinoblastoma gene family pRb/p105, p107, pRb2/p130 and simian virus-40 large T-antigen in human mesotheliomas. *Nature Med* **3**: 913–916

De Marzo AM, Coffey DS and Nelson WG (1999) New concepts in tissue specificity for prostate cancer and benign prostatic hyperplasia. *Urology* **53**: 29–42

del Peso L, Gonzalez-Garcia M, Page C, Herrera R and Nunez G (1997) Interleukin-3-induced phosphorylation of BAD through the protein kinase Akt. *Science* **278**: 687–689

Della Bella S, Molteni M, Compasso S, Zulian C, Vanoli M and Scorza R (1997) Differential effects of cyclo-oxygenase pathway metabolites on cytokine production by T lymphocytes. *Prostaglandins Leukot Essent Fatty Acids* **56**: 177–184

Di Popolo A, Memoli A, Apicella A, Tuccillo C, di Palma A, Ricchi P, Acquaviva AM and Zarrilli R (2000) IGF-II/IGF-I receptor pathway up-regulates COX-2 mRNA expression and PGE2 synthesis in Caco-2 human colon carcinoma cells. *Oncogene* **19**: 5517–5524

Doherty PC, Tripp RA and Sixbey JW (1994) Evasion of host immune responses by tumours and viruses. *Ciba Found Symp* **187**: 245–256

DuBois RN, Abramson SB, Crofford L, Gupta RA, Simon LS, Van de Putte LBA and Lipsky PE (1998) Cyclooxygenase in biology and disease. *FASEB J* **12**: 1063–1073

Dumas F, Eschwege P and Loric S (1997) Acute bacterial prostatitis induces hematogenous dissemination of prostate epithelial cells [letter]. *Clin Chem* **43**: 2007–2008

Duperron C and Castonguay A (1997) Chemopreventive efficacies of aspirin and sulindac against lung tumorigenesis in A/J mice. *Carcinogenesis* **18**: 1001–1006

Edwards JG, Abrams KR, Leverment JN, Spyt TJ, Waller DA and O'Byrne KJ (2000) Prognostic factors for malignant mesothelioma in 142 patients: validation of CALGB and EORTC prognostic scoring systems. *Thorax* **55**: 731–735

El-Bayoumy KIM, Amin S, Hoffman D and Wynder EL (1999) Increased expression of cyclooxygenase-2 in rat lung tumours induced by the tobacco-specific nitrosamine 4-(methylnitrosamino)-4-(3-pyridyl)-1-butanone: the impact of a high-fat diet. *Cancer Research* **59**: 1400–1403

Eliopoulos AG, Gallagher NJ, Blake SM, Dawson CW and Young LS (1999) Activation of the p38 mitogen-activated protein kinase pathway by Epstein-Barr virus-encoded latent membrane protein 1 coregulates interleukin-6 and interleukin-8 production. *J Biol Chem* **274**: 16085–16096

Faux SP and Howden PJ (1997) Possible role of lipid peroxidation in the induction of NF-kB and AP-1 in RFL-6 cells by crocidolite asbestos: evidence following protection by vitamin E. *Environ Health Perspect* **105**: 1127–1130

Faux SP, Houghton CE, Hubbard A and Patrick G (2000) Increased expression of epidermal growth factor receptor in rat pleural mesothelial cells correlates with carcinogenicity of mineral fibres. *Carcinogenesis* **21**: 2275–2280

Faux SP, Houghton CE, Swain WA, Edwards JG, Sharma RA, Plummer SM and O'Byrne KJ (2001) EGFR induced activation of NF-kB in mesothelial cells by asbestos is important in cell survival. *Proc Am Assoc Cancer Res*: abstract 1315

Fleming S (1999) Renal cancer genetics: von Hippel Lindau and other syndromes.

 © 2001 Cancer Research Campaign

*Int J Dev Biol* **43**: 469–471

Folkman J (1995) Seminars in Medicine of the Beth Israel Hospital, Boston. Clinical applications of research on angiogenesis [see comments]. *N Engl J Med* **333**: 1757–1763

Funkhouser EM and Sharp GB (1995) Aspirin and reduced risk of esophageal carcinoma. *Cancer* **76**: 1116–1169

Gabrilovich DI, Ishida T, Nadaf S, Ohm JE and Carbone DP (1999) Antibodies to vascular endothelial growth factor enhance the efficacy of cancer immunotherapy by improving endogenous dendritic cell function. *Clin Cancer Res* **5**: 2963–2970

Gallucci RM, Simeonova PP, Matheson JM, Kommineni C, Guriel JL, Sugawara T and Luster MI (2000) Impaired cutaneous wound healing in interleukin-6-deficient and immunosuppressed mice. *FASEB J* **14**: 2525–2531

Ganss R and Hanahan D (1998) Tumor microenvironment can restrict the effectiveness of activated antitumor lymphocytes. *Cancer Res* **58**: 4673–4681

Garrido F, Cabrera T, Concha A, Glew S, Ruiz-Cabello F and Stern PL (1993) Natural history of HLA expression during tumour development. *Immunol Today* **14**: 491–499

Gately S (2000) The contributions of cyclooxygenase-2 to tumor angiogenesis. *Cancer Metastasis Rev* **19**: 19–27

Giovannucci E, Egan KM, Hunter DJ, Stampfer MJ, Colditz GA, Willett WC and Speizer FE (1995) Aspirin and the risk of colorectal cancer in women [see comments]. *N Engl J Med* **333**: 609–614

Giovannucci E, Rimm EB, Stampfer MJ, Colditz GA, Ascherio A and Willett WC (1994) Aspirin use and the risk for colorectal cancer and adenoma in male health professionals. *Ann Intern Med* **121**: 241–246

Gjertsen MK, Bjorheim J, Saeterdal I, Myklebust J and Gaudernack G (1997) Cytotoxic CD4+ and CD8+ T lymphocytes, generated by mutant p21-ras (12Val) peptide vaccination of a patient, recognize 12Val-dependent nested epitopes present within the vaccine peptide and kill autologous tumour cells carrying this mutation. *Int J Cancer* **72**: 784–790

Gorter A and Meri S (1999) Immune evasion of tumor cells using membrane-bound complement regulatory proteins. *Immunol Today* **20**: 576–582

Grinwich KD and Plescia OJ (1977) Tumor-mediated immunosuppression: prevention by inhibitors of prostaglandin synthesis. *Prostaglandins* **14**: 1175–1182

Guillou L, Estreicher A, Chaubert P, Hurlimann J, Kurt AM, Metthez G, Iggo R, Gray AC, Jichlinski P, Leisinger HJ and Benhattar J (1996) Germ cell tumors of the testis overexpress wild-type p53. *Am J Pathol* **149**: 1221–1228

Habeshaw J, Hounsell E and Dalgleish A (1992) Does the HIV envelope induce a chronic graft-versus-host-like disease? *Immunol Today* **13**: 207–210

Heriot AG, Marriott JB, Cookson S, Kumar D and Dalgleish AG (2000) Reduction in cytokine production in colorectal cancer patients: association with stage and reversal by resection. *Br J Cancer* **82**: 1009–1012

Hida TLJ, Makheja AN, Ben-Av P, Hla T, Martinez A, Mulshine J, Malkani S, Chung P and Moody TW (1998) Non-small cell lung cancer cycloxygenase activity and proliferation are inhibited by non-steroidal antiinflammatory drugs. *Anticancer Res* **18**: 775–782

Higaki S, Akazawa A, Nakamura H, Yanai H, Yoshida T and Okita K (1999) Metaplastic polyp of the colon develops in response to inflammation. *J Gastroenterol Hepatol* **14**: 709–714

Hildesheim A, Schiffman MH, Tsukui T, Swanson CA, Lucci J, Scott DR, Glass AG, Rush BB, Lorincz AT, Corrigan A, Burk RD, Helgesen K, Houghten RA, Sherman ME, Kurman RJ, Berzofsky JA and Kramer TR (1997) Immune activation in cervical neoplasia: cross-sectional association between plasma soluble interleukin 2 receptor levels and disease. *Cancer Epidemiol Biomarkers Prev* **6**: 807–813

Hopfl R, Heim K, Christensen N, Zumbach K, Wieland U, Volgger B, Widschwendter A, Haimbuchner S, Muller-Holzner E, Pawlita M, Pfister H and Fritsch P (2000) Spontaneous regression of CIN and delayed-type hypersensitivity to HPV-16 oncoprotein E7. *Lancet* **356**: 1985–1986

Horikawa T, Yoshizaki T, Sheen TS, Lee SY and Furukawa M (2000) Association of latent membrane protein 1 and matrix metalloproteinase 9 with metastasis in nasopharyngeal carcinoma. *Cancer* **89**: 715–723

Hsieh CY, You SL, Kao CL and Chen CJ (1999) Reproductive and infectious risk factors for invasive cervical cancer in Taiwan. *Anticancer Res* **19**: 4495–4500

Hu P, Margolis B, Skolnik EY, Lammers R, Ullrich A and Schlessinger J (1992) Interaction of phosphatidylinositol 3-kinase-associated p85 with epidermal growth factor and platelet-derived growth factor receptors. *Mol Cell Biol* **12**: 981–990

Huang M, Stolina M, Sharma S, Mao JT, Zhu L, Miller PW, Wollman J, Herschman H and Dubinett SM (1998) Non-small cell lung cancer cyclooxygenase-2-dependent regulation of cytokine balance in lymphocytes and macrophages: up-regulation of interleukin 10 and down-regulation of interleukin 12 production. *Cancer Res* **58**: 1208–1216

Huang YT, Sheen TS, Chen CL, Lu J, Chang Y, Chen JY and Tsai CH (1999) Profile of cytokine expression in nasopharyngeal carcinomas: a distinct expression of interleukin 1 in tumor and CD4+ T cells. *Cancer Res* **59**: 1599–1605

Hudson JD, Shoaibi MA, Maestro R, Carnero A, Hannon GJ and Beach DH (1999) A proinflammatory cytokine inhibits p53 tumor suppressor activity. *J Exp Med* **190**: 1375–1382

Imperial JC (1999) Natural history of chronic hepatitis B and C. *J Gastroenterol Hepatol* **14**: Suppl S1–S5

Irani J, Goujon JM, Ragni E, Peyrat L, Hubert J, Saint F and Mottet N (1999) High-grade inflammation in prostate cancer as a prognostic factor for biochemical recurrence after radical prostatectomy. Pathologist Multi Center Study Group. *Urology* **54**: 467–472

Jacobs N, Giannini SL, Doyen J, Baptista A, Moutschen M, Boniver J and Delvenne P (1998) Inverse modulation of IL-10 and IL-12 in the blood of women with preneoplastic lesions of the uterine cervix. *Clin Exp Immunol* **111**: 219–224

Jankowski JA, Wright NA, Meltzer SJ, Triadafilopoulos G, Geboes K, Casson AG, Kerr D and Young LS (1999) Molecular evolution of the metaplasia-dysplasia-adenocarcinoma sequence in the esophagus. *Am J Pathol* **154**: 965–973

Janssen YM, Driscoll KE, Howard B, Quinlan TR, Treadwell M, Barchowsky A and Mossman BT (2000) Asbestos causes translocation of p65 protein and increases NF-kappaB DNA binding activity in rat lung epithelial and pleural mesothelial cells. *Am J Pathol* **151**: 389–401

Jung K, Meyer A, Lein M, Rudolph B, Schnorr D and Loening SA (1998) Ratio of free-to-total prostate specific antigen in serum cannot distinguish patients with prostate cancer from those with chronic inflammation of the prostate. *J Urol* **159**: 1595–1598

Kano A, Watanabe Y, Takeda N, Aizawa S and Akaike T (1997) Analysis of IFN-gamma induced cell cycle arrest and cell death in hepatocytes. *J Biochemistry* **121**: 677–683

Keay S, Zhang CO, Baldwin BR and Alexander RB (1999) Polymerase chain reaction amplification of bacterial 16s rRNA genes in prostate biopsies from men without chronic prostatitis. *Urology* **53**: 487–491

Kirk GR and Clements WD (1999) Crohn's disease and colorectal malignancy. *Int J Clin Pract* **53**: 314–315

Kitagawa N, Goto M, Kurozumi K, Maruo S, Fukayama M, Naoe T, Yasukawa M, Hino K, Suzuki T, Todo S and Takada K (2000) Epstein-Barr virus-encoded poly(A)(-) RNA supports Burkitt's lymphoma growth through interleukin-10 induction. *EMBO J* **19**: 6742–6750

Kodelja V, Muller C, Tenorio S, Schebesch C, Orfanos CE and Goerdt S (1997) Differences in angiogenic potential of classically vs alternatively activated macrophages. *Immunobiology* **197**: 478–493

Konturek PC, Hartwich A, Zuchowicz M, Labza H, Pierzchalski P, Karczewska E, Bielanski W, Hahn EG and Konturek SJ (2000) Helicobacter pylori, gastrin and cyclooxygenases in gastric cancer. *J Physiol Pharmacol* **51**: 737–749

Koshiba T, Hosotani R, Miyamoto Y, Wada M, Lee JU, Fujimoto K, Tsuji S, Nakajima S, Doi R and Imamura M (1999) Immunohistochemical analysis of cyclooxygenase-2 expression in pancreatic tumors. *Int J Pancreatol* **26**: 69–76

Lala PK, Al-Mutter N and Orucevic A (1997) Effects of chronic indomethacin therapy on the development and progression of spontaneous mammary tumors in C3H/HEJ mice. *Int J Cancer* **73**: 371–380

Langman MJ, Cheng KK, Gilman EA and Lancashire RJ (2000) Effect of anti-inflammatory drugs on overall risk of common cancer: case-control study in general practice research database. *BMJ* **320**: 1642–1646

Le Buanec H, D'Anna R, Lachgar A, Zagury JF, Bernard J, Ittele D, d'Alessio P, Hallez S, Giannouli C, Burny A, Bizzini B, Gallo RC and Zagury D (1999) HPV-16 E7 but not E6 oncogenic protein triggers both cellular immunosuppression and angiogenic processes. *Biomed Pharmacother* **53**: 424–431

Lee PP, Zeng D, McCaulay AE, Chen YF, Geiler C, Umetsu DT and Chao NJ (1997) T helper 2-dominant antilymphoma immune response is associated with fatal outcome. *Blood* **90**: 1611–1617

Lengauer C, Kinzler KW and Vogelstein B (1998) Genetic instabilities in human cancers. *Nature* **396**: 643–649

Lewis JD, Deren JJ and Lichtenstein GR (1999) Cancer risk in patients with inflammatory bowel disease. *Gastroenterol Clin North Am* **28**: 459–477

Liu MY, Shih YY, Li LY, Chou SP, Sheen TS, Chen CL, Yang CS and Chen JY (2000) Expression of the Epstein-Barr virus BHRF1 gene, a homologue of Bcl-2, in nasopharyngeal carcinoma tissue. *J Med Virol* **61**: 241–250

Majima M, Isono M, Ikeda Y, Hayashi I, Hatanaka K, Harada Y, Katsumata O, Yamashina S, Katori M and Yamamoto S (1997) Significant roles of inducible cyclooxygenase (COX)-2 in angiogenesis in rat sponge implants. *Jpn J Pharmacol* **75**: 105–114

Maraveyas A, Baban B, Kennard D, Rook GA, Westby M, Grange JM, Lydyard P,

*© 2001 Cancer Research Campaign* 

Stanford JL, Jones M, Selby P and Dalgleish AG (1999) Possible improved survival of patients with stage IV AJCC melanoma receiving SRL 172 immunotherapy: correlation with induction of increased levels of intracellular interleukin-2 in peripheral blood lymphocytes. *Ann Oncol* **10**: 817–824

Mauray S, Fuzzati-Armentero MT, Trouillet P, Ruegg M, Nicoloso G, Hart M, Aarden L, Schapira M and Duchosal MA (2000) Epstein-Barr virus-dependent lymphoproliferative disease: critical role of IL-6. *Eur J Immunol* **30**: 2065–2073

Mayne ST, Buenconsejo J and Janerich DT (1999). Previous lung disease and risk of lung cancer among men and women nonsmokers. *Am J Epidemiol* **149**: 13–20

McCann J (1999) Esophageal cancers: changing character, increasing incidence [news]. *J Natl Cancer Inst* **91**: 497–498

McGinty A, Chang YW, Sorokin A, Bokemeyer D and Dunn MJ (2000) Cyclooxygenase-2 expression inhibits trophic withdrawal apoptosis in nerve growth factor-differentiated PC12 cells. *J Biol Chem* **275**: 12095–12101

Melief CJ and Kast WM (1991) Cytotoxic T lymphocyte therapy of cancer and tumor escape mechanisms. *Semin Cancer Biol* **2**: 347–354

Mestre JR, Chan G, Zhang F, Yang EK, Sacks PG, Boyle JO, Shah JP, Edelstein D, Subbaramaiah K and Dannenberg AJ (1999) Inhibition of cyclooxygenase-2 expression. An approach to preventing head and neck cancer. *Ann N Y Acad Sci* **889**: 62–71

Molina MA, Sitja-Arnau M, Lemoine MG, Frazier ML and Sinicrope FA (1999) Increased cyclooxygenase-2 expression in human pancreatic carcinomas and cell lines: growth inhibition by nonsteroidal anti-inflammatory drugs. *Cancer Res* **59**: 4356–4362

Monson RR, Yen S and MacMahon B (1976) Chronic mastitis and carcinoma of the breast. *Lancet* **2**: 224–226

Moore RJ, Owens DM, Stamp G, Arnott C, Burke F, East N, Holdsworth H, Turner L, Rollins B, Pasparakis M, Kollias G and Balkwill F (1999) Tumour necrosis factor-a deficient mice are resistant to skin carcinogenesis. *Nat Med* **5**: 828–831

Mosmann TR and Coffman RL (1989) TH1 and TH2 cells: different patterns of lymphokine secretion lead to different functional properties. *Annu Rev Immunol* **7**: 145–173

Murata H, Kawano S, Tsuji S, Tsuji M, Sawaoka H, Kimura Y, Shiozaki H and Hori M (1999) Cyclooxygenase-2 overexpression enhances lymphatic invasion and metastasis in human gastric carcinoma. *Am J Gastroenterol* **94**: 451–455

Nakata S, Imai K and Yamanaka H (1993) Study of risk factors for prostatic cancer. *Hinyokika Kiyo* **39**: 1017–1024

O'Byrne KJ, Dalgleish AG, Browning MJ, Steward WP and Harris AL (2000) The relationship between angiogenesis and the immune response in carcinogenesis and the progression of malignant disease. *Eur J Cancer* **36**: 151–169

Paganini-Hill A (1994) Aspirin and the prevention of colorectal cancer: a review of the evidence. *Semin Surg Oncol* **10**: 158–164

Pamphilon DH, Alnaqdy AA and Wallington TB (1991) Immunomodulation by ultraviolet light: clinical studies and biological effects. *Immunol Today* **12**: 119–123

Pettit SJ, Seymour K, O'Flaherty E and Kirby JA (2000) Immune selection in neoplasia: towards a microevolutionary model of cancer development. *Br J Cancer* **82**: 1900–1906

Peus D, Vasa RA, Meves A, Beyerle A and Pittelkow MR (2000) UVB-induced epidermal growth factor receptor phosphorylation is critical for downstream signaling and keratinocyte survival. *Photochem Photobiol* **72**: 135–140

Piccinni MP, Beloni L, Livi C, Maggi E, Scarselli G and Romagnani S (1998) Defective production of both leukemia inhibitory factor and type 2 T-helper cytokines by decidual T cells in unexplained recurrent abortions. *Nat Med* **4**: 1020–1024

Prince MM and Hildreth NG (1986) The influence of potential biases on the risk of breast tumors among women who received radiotherapy for acute postpartum mastitis. *J Chronic Dis* **39**: 553–560

Raza A (2000) Consilience across evolving dysplasias affecting myeloid, cervical, esophageal, gastric and liver cells: common themes and emerging patterns. *Leuk Res* **24**: 63–72

Raziuddin S, Shetty S and Ibrahim A (1991) T-cell abnormality and defective interleukin-2 production in patients with carcinoma of the urinary bladder with schistosomiasis. *J Clin Immunol* **11**: 103–113

Richards JS, Fitzpatrick SL, Clemens JW, Morris JK, Alliston T and Sirois J (1995) Ovarian cell differentiation: a cascade of multiple hormones, cellular signals, and regulated genes. *Recent Prog Horm Res* **50**: 223–254

Rook GA, Hernandez-Pando R and Lightman SL (1994) Hormones, peripherally activated prohormones and regulation of the Th1/Th2 balance. *Immunol Today* **15**: 301–303

Saareks V, Mucha I, Sievi E, Vapaatalo H and Riutta A (1998) Nicotine stereoisomers and cotinine stimulate prostaglandin E2 but inhibit thromboxane B2 and leukotriene E4 synthesis in whole blood. *Eur J Pharmacol* **353**: 87–92

Savanthanan S and O'Byrne KJ (2001) Antiestrogens and breast cancer. *J Br Meno Soc* **7**: 21–26

Sawaoka H, Kawano S, Tsuji S, Tsujii M, Sun W, Gunawan ES and Hori M (1998a) Helicobacter pylori infection induces cyclooxygenase-2 expression in human gastric mucosa. *Prostaglandins Leukot Essent Fatty Acids* **59**: 313–316

Sawaoka H, Kawano S, Tsuji S, Tsujii M, Gunawan ES, Takei Y, Nagano K and Hori M (1998b) Cyclooxygenase-2 inhibitors suppress the growth of gastric cancer xenografts via induction of apoptosis in nude mice. *Am J Physiol* **274**: G1061–G1067

Sawaoka H, Kawano S, Tsuji S, Tsujii M, Murata H and Hori M (1998c) Effects of NSAIDs on proliferation of gastric cancer cells in vitro: possible implication of cyclooxygenase-2 in cancer development. *J Clin Gastroenterol* **27**: S47–S52

Sawaoka H, Tsuji S, Tsujii M, Gunawan ES, Sasaki Y, Kawano S and Hori M (1999) Cyclooxygenase inhibitors suppress angiogenesis and reduce tumor growth in vivo. *Lab Invest* **79**: 1469–1477

Schaffer M and Barbul A (1998) Lymphocyte function in wound healing and following injury. *Br J Surg* **85**: 444–460

Schreinemachers DM and Everson RB (1994) Aspirin use and lung, colon, and breast cancer incidence in a prospective study. *Epidemiology* **5**: 138–146

Schror K, Zimmermann KC and Tannhauser R (1998) Augmented myocardial ischaemia by nicotine–mechanisms and their possible significance. *Br J Pharmacol* **125**: 79–86

Scott M, Stites DP and Moscicki AB (1999) Th1 cytokine patterns in cervical human papillomavirus infection. *Clin Diagn Lab Immunol* **6**: 751–755

Singer AJ and Clark RA (1999) Cutaneous wound healing. *N Engl J Med* **341**: 738–746

Spitz MR, Wei Q, Li G and Wu X (1999) Genetic susceptibility to tobacco carcinogenesis. *Cancer Invest* **17**: 645–659

Spivak MYA, Lakatosh VP, Lazarenko LM, Lyanenko LM, Azarskova MV, Mikhailenko OM, Tkacikova L and Boroda AM (1999) Interrelation of lymphocyte subpopulations in peripheral blood under cervical papillomavirus infection. *Folia Microbiol (Praha)* **44**: 721–725

Strand S and Galle PR (1998) Immune evasion by tumours: involvement of the CD95 (APO-1/Fas) system and its clinical implications. *Mol Med Today* **4**: 63–68

Study C (1992) The American Cancer Society Prospective Study. *Stat Bull Metrop Issue Co* **73**: 21–29

Subbaramaiah K, Zakim D, Weksler BB and Dannenberg AJ (1997) Inhibition of cyclooxygenase: a novel approach to cancer prevention. *Proc Soc Exp Biol Med* **216**: 201–210

Takada K (2000) Epstein-Barr virus and gastric carcinoma. *Mol Pathol* **53**: 255–261

Takahashi Y, Kawahara F, Noguchi M, Miwa K, Sato H, Seiki M, Inoue H, Tanabe T and Yoshimoto T (1999) Activation of matrix metalloproteinase-2 in human breast cancer cells overexpressing cyclooxygenase-1 or -2. *FEBS Lett* **460**: 145–148

Taketo MM (1998) Cyclooxygenase-2 inhibitors in tumorigenesis (Part II). *J Natl Cancer Inst* **90**: 1609–1620

Thanos D and Maniatis T (2000) NF-kB: a lesson in family values. *Cell* **80**: 529–532

Thun MJ, Namboodiri MM and Health CW Jr (1991) Aspirin use and reduced risk of fatal colon cancer. *N Engl J Med* **325**: 1593–1596

Tomozawa S, Nagawa H, Tsuno N, Hatano K, Osada T, Kitayama J, Sunami E, Nita ME, Ishihara S, Yano H, Tsuruo T, Shibata Y and Muto T (1999) Inhibition of haematogenous metastasis of colon cancer in mice by a selective COX-2 inhibitor, JTE-522. *Br J Cancer* **81**: 1274–1279

Tsuji S, Kawano S, Sawaoka H, Takei Y, Kobayashi I, Nagano K, Fusamoto H and Kamada T (1996) Evidences for involvement of cyclooxygenase-2 in proliferation of two gastrointestinal cancer cell lines. *Prostaglandins Leukot Essent Fatty Acids* **55**: 179–183

Tsujii M and DuBois RN (1995) Alterations in cellular adhesion and apoptosis in epithelial cells overexpressing prostaglandin endoperoxide synthase 2. *Cell* **83**: 493–501

Tsujii M, Kawano S and DuBois RN (1997) Cyclooxygenase-2 expression in human colon cancer cells increases metastatic potential. *Proc Natl Acad Sci U S A* **94**: 3336–3340

Tsujii M, Kawano S, Tsuji S, Sawaoka H, Hori M and DuBois RN (1998) Cyclooxygenase regulates angiogenesis induced by colon cancer cells. *Cell* **93**: 705–716

Ung A, Kramer TR, Schiffman M, Herrero R, Bratti MC, Burk RD, Swanson CA, Sherman ME, Hutchinson ML, Alfaro M, Morales J, Balmaceda I and Hildesheim A (1999) Soluble interleukin 2 receptor levels and cervical neoplasia: results from a population-based case-control study in Costa Rica. *Cancer Epidemiol Biomarkers Prev* **8**: 249–253

Uotila P (1996) The role of cyclic AMP and oxygen intermediates in the inhibition

© 2001 Cancer Research Campaign of cellular immunity in cancer. *Cancer Immunol Immunother* **43**: 1–9
Vaganova IG (2000) Apoptosis and proliferation of epithelial cells in papillomaviral and chlamydial cervicitis. *Vopr Onkol* **46**: 578–582
Vainio H and Morgan G (1998) Cyclo-oxygenase 2 and breast cancer prevention. Non-steroidal anti-inflammatory agents are worth testing in breast cancer [editorial]. *B M J* **317**: 828
Vane JR, Bakhle YS and Botting RM (1998) Cyclooxygenases 1 and 2. *Annu Rev Pharmacol Toxicol* **38**: 97–120
Vile RSB and Dalgleish AG (1996) *Tumour Vaccines*. In: *Immunotherapy in Cancer*. John Wiley & Sons Ltd: Chichester
Vogelstein B, Fearon ER, Hamilton SR, Kern SE, Preisinger AC, Leppert M, Nakamura Y, White R, Smits AM and Bos JL (1988) Genetic alterations during colorectal-tumor development. *N Engl J Med* **319**: 525–532
Wallenius V, Wallenius K and Jansson JO (2000) Normal pharmacologically-induced, but decreased regenerative liver growth in interleukin-6-deficient (IL-6-(-/-)) mice. *J Hepatol* **233**: 967–974
Wang LS, Chow KC, Wu YC, Li WY and Huang MH (1999) Detection of Epstein-Barr virus in esophageal squamous cell carcinoma in Taiwan. *Am J Gastroenterol* **94**: 2834–2839
Watanabe M, McCormick KL, Volker K, Ortaldo JR, Wigginton JM, Brunda MJ, Wiltrout RH and Fogler WE (1997) Regulation of local host-mediated anti-tumor mechanisms by cytokines: direct and indirect effects on leukocyte recruitment and angiogenesis. *Am J Pathol* **150**: 1869–1880
Weiss RDA and Loveday C (1999) *Human Immunodeficincy Viruses*. In: Vol. 44. *Principles and Practice of Clinical Virology*. Wiley: Location
Westby M, Marriott JB, Guckian M, Cookson S, Hay P and Dalgleish AG (1998) Abnormal intracellular IL-2 and interferon-gamma (IFN-gamma) production as HIV-1-assocated markers of immune dysfunction. *Clin Exp Immunol* **111**: 257–263
Whitby D and Boshoff C (1998) Kaposi's sarcoma herpesvirus as a new paradigm for virus-induced oncogenesis. *Curr Opin Oncol* **10**: 405–412
White CD, Macatol FR and DeJosef AB (1992) Inflammatory cell infiltrate in the cervix as a predictor of residual cervical intraepithelial neoplasia after conization. *J Reprod Med* **37**: 799–802
Williams MP and Pounder RE (1999) Helicobacter pylori: from the benign to the malignant. *Am J Gastroenterol* **94**: S11–S16
Wolff H, Saukkonen K, Anttila S, Karjalainen A, Vainio H and Ristimaki A (1998) Expression of cyclooxygenase-2 in human lung carcinoma. *Cancer Res* **58**: 4997–5001
Xu Z-G, Iwatsuki K, Oyama N, Ohtsuka M, Satoh M, Kikuchi S, Akiba H and Kaneko F (2001) The latency pattern of Epstein-Barr virus infection and viral IL-10 expression in cutaneous natural killer/T-cell lymphomas. *Br J Cancer* **84**: 920–925
Yamamoto T, Nakamura Y, Kishimoto K, Takeuchi H, Shirakata M, Mitsuya T and Hirai K (1999) Epstein-Barr virus (EBV)-infected cells were frequently but dispersely detected in T-cell lymphomas of various types by in situ hybridization with an RNA probe specific to EBV-specific nuclear antigen 1. *Virus Res* **65**: 43–55
Yang T, Schnermann JB and Briggs JP (1999) Regulation of cyclooxygenase-2 expression in renal medulla by tonicity in vivo and in vitro. *Am J Physiol* **277**: F1–F9
Yao R, Rioux N, Castonguay A and You M (2000) Inhibition of COX-2 and induction of apoptosis: two determinants of nonsteroidal anti-inflammatory drugs' chemopreventive efficacies in mouse lung tumorigenesis. *Exp Lung Res* **26**: 731–742
Yeung MC and Lau AS (1998) Tumor suppressor p53 as a component of the tumor necrosis factor-induced, protein kinase PKR-mediated apoptotic pathway in human promonocytic U937 cells. *J Biol Chem* **273**: 25198–25202
Yucel-Lindberg T, Ahola H, Carlstedt-Duke J and Modeer T (1999) Involvement of tyrosine kinases on cyclooxygenase expression and prostaglandin E2 production in human gingival fibroblasts stimulated with interleukin-1beta and epidermal growth factor. *Biochem Biophys Res Commun* **257**: 528–532
Zhou BP, Hu M, Hu CT, Miller SA, Yu Z, Xia W, Lin S-Y and Hung M-C (2000) HER-2/*neu* blocks tumour necrosis factor-induced apoptosis via the Akt/NF-kB pathway. *J Biol Chem* **275**: 8027–8031
zur Hausen A, Brink AA, Craanen ME, Middeldorp JM, Meijer CJ and van den Brule AJ (2000) Unique transcription pattern of Epstein-Barr virus (EBV) in EBV-carrying gastric adenocarcinomas: expression of the transforming BARF1 gene. *Cancer Res* **60**: 2745–2748

*© 2001 Cancer Research Campaign*
